# Babies

**Published:** 1860-12

**Authors:** 


### HALL'S JOURNAL OF HEALTH.

Our Legitimate Scope is almost boundless: for whatever begets pleasurable and harmless feelings, promotes Health; and whatever induces disagreeable sensations, engenders Disease.

WE AIM TO SHOW HOW DISEASE MAY BE AVOIDED, AND THAT IT IS BEST, WHEN SICKNESS COMES, TO TAKE NO MEDICINE WITHOUT CONSULTING A PHYSICIAN.

Vol. VII.] DECEMBER, 1860. [No. 12.

### BABIES.

SOME Rev. Benedictine is ventilating himself through the papers, on the subject of "Baby Talk." He mounts on stilts forty feet high, and then lowers himself by using such strong words as "detestable," "unjust," "ridiculous," "distorted," "mangled," "burlesque," "barbarized," etc. Now, who but a crusty old "bach" could look at a sweet little child, and then go off into such a diarrhea of sweeping adjectives, not one of which can be thought of without feelings akin to those associ ated with a mouthful of vinegar. He thinks a great wrong is done a little prattler by teaching it to say "Horsey" and "Mudder." And to call a dog "bow-wow," is awful! He is only mad because he couldn't raise a baby himself, and wants to put a "spider in the dumpling" of those who have a house full of the dear, delightful responsibilities. Only hear the man: "This seems ridiculous, but that is not all, it is unjust to teach pronunciations which he must unlearn, as laboriously as they were learned. You thus double the task. The folly and injus. tice are the same, when you teach a little child to speak a distorted, mangled, burlesque language,.of which, when older, it becomes ashamed. I object to this clipped and barbarous Eng. lish, because it involves a waste of time, and brain power, and patience." Surely this man is snuffing the wind. He must have been in a highly imaginative mood when he wrote those lines, or the east wind was blowing, or he had a fit of dyspepsia. Perhaps he had just received a "mitten." At all events, his mental vision was considerably obfuscated or preternaturally brightened, since

"Optics sharp, it needs, I ween!
To see what is not to be seen."

We indite this article for the special benefit of Babydom for now and all time, and desire to crush the error in the bud; and these are the reasons:

It will not be denied that the most natural language in the world, and the most easily learned, is that whose words express the most characteristic quality of the thing named. The rumbling of the thunder, the hissing of a snake, the barking of a dog in the bow-wow, are associated in name and nature. It must be manyfold easier for a child to connect bow-wow with a dog, after the first heard bark, than with the word "dog." It can see the connection in the former case, and the memory is aided by the association; in fact, it requires but an instinctive effort of the memory; while to connect "dog," with the noise it makes, requires an abstract effort of the memory, which is burdensome, and in mature life we all avoid it when we can, by thinking of a familiar thing, with a view to its connection with something less familiar, which is desired to be remembered.

The same may be said as to the word mother. It is much easier for the lisping child to say "mudder," for it has not acquired that facility of tongue and lip movement which is necessary to a distinct pronunciation of the dear name. In fact, it is simply an impossibility for a child just learning to talk, to say "mother." A child must toddle before walking; it must also toddle before talking; and it requires no more effort to talk better, than to walk better; both abilities come to them so gradually and so naturally, as the muscles of the parts become more flexible and under control, that in neither case is there a consciousness of effort. A man must learn the pronunciation of a language foreign to his own, whether living or dead, by degrees; and to require a faultless pronunciation from the first, is an unnecessary infliction—it can not be done. The ear must gradually learn the niceities of pronunciation by frequent hearing, and the lips and tongue must be adjusted accordingly.

Again, all languages have forms of expression which signify endearment or intensification. In the English it seems to be a kind of a rhyme, such as Horsey-porsy, Piggy-wiggy, Georgy-porgy, Lijah-pygy. Besides, a close observer may see that it is easier to pronounce a word ending with y, than one which has none; just as it is easier to stop by degrees, than short off. It is easier to say Horsey, than a clear short "Horse."

The fact is, a man can't talk dictionary himself, without piling up the dignity; and why should a parent care a fig about dignity, when he is melting away under the softening influences of childhood's sunshine? It's only "stuck-up" people who are everlastingly retreating on their own proprieties. It requires a Pitt to play marbles with his boy; a Napoleon to be on all-fours, with his child astride of his back, to be swept off on the floor by the biped horse running under the tables. They are wise who can be children twice; who can bend at pleasure from age to infancy. There is no incompatibility between firmness and love; between stately dignity and an affectionate heart. A parent's presence should carry with it the gladdening sunshine, and not the chilling iceberg. So, dear reader, if you are so happy as to have children, do not mar it when you are with them, by mounting stilts or talking dictionary; throw off your corsets, make yourself "one of them," and be assured, you and they will be the happier thereby; the Rev. J. T. Benedict D.D., to the contrary notwithstanding.

